# Combination of Hemoglobin-for-Age Z-Score and Plasma Hepcidin Identified as a Novel Predictor for Kawasaki Disease

**DOI:** 10.3390/children9060913

**Published:** 2022-06-18

**Authors:** Ya-Ling Yang, Ho-Chang Kuo, Kuang-Den Chen, Chi-Hsiang Chu, Kuang-Che Kuo, Mindy Ming-Huey Guo, Ling-Sai Chang, Ying-Hsien Huang

**Affiliations:** 1 Department of Anesthesiology, Kaohsiung Chang Gung Memorial Hospital and Chang Gung University College of Medicine, Kaohsiung 833, Taiwan; inr453@cgmh.org.tw; 2 Kawasaki Disease Center, Kaohsiung Chang Gung Memorial Hospital and Chang Gung University College of Medicine, Kaohsiung 833, Taiwan; erickuo48@yahoo.com.tw (H.-C.K.); light@cgmh.org.tw (K.-C.K.); mindymhguo@yahoo.com.tw (M.M.-H.G.); joycejohnsyoko@gmail.com (L.-S.C.); 3 Department of Pediatrics, Kaohsiung Chang Gung Memorial Hospital and Chang Gung University College of Medicine, Kaohsiung 833, Taiwan; 4 Institute for Translational Research in Biomedicine, Kaohsiung Chang Gung Memorial Hospital and Chang Gung University College of Medicine, Kaohsiung 833, Taiwan; dennis8857@gmail.com; 5 Liver Transplantation Center, Department of Surgery, Kaohsiung Chang Gung Memorial Hospital and Chang Gung University College of Medicine, Kaohsiung 833, Taiwan; 6 Department of Statistics, Tunghai University, Taichung 407, Taiwan; loveweib@gmail.com

**Keywords:** Kawasaki disease, score system, child hemoglobin-for-age z-score, hepcidin

## Abstract

Kawasaki disease (KD) is a febrile coronary vasculitis that affects younger children and includes complications such as coronary artery aneurysm. KD diagnoses are diagnosed based on clinical presentations, a process that still poses a challenge for front-line physicians. In the current study, we developed a novel predictor using the hemoglobin-for-age z-score (HbZ) and plasma hepcidin to differentiate Kawasaki disease (KD) from febrile children (FC). There were 104 FC and 115 KD subjects (89 typical KD; 26 incomplete KD) for this study, and data were collected on the biological parameters of hemoglobin and plasma hepcidin levels. A receiver operating characteristic curve (auROC), multiple logistics regression, and support vector machine analysis were all adopted to develop our prediction condition. We obtained both predictors, HbZ and plasma hepcidin, for distinguishing KD and FC. The auROC of the multivariate logistic regression of both parameters for FC and KD was 0.959 (95% confidence interval = 0.937–0.981), and the sensitivity and specificity were 85.2% and 95.9%, respectively. Furthermore, the auROC for FC and incomplete KD was 0.981, and the sensitivity and specificity were 92.3% and 95.2%, respectively. We further developed a model of support vector machine (SVM) classification with 83.3% sensitivity and 88.0% specificity in the training set, and the blind cohort performed well (78.4% sensitivity and 100% specificity). All data showed that sensitivity and specificity were 81.7% and 91.3%, respectively, by SVM. Overall, our findings demonstrate a novel predictor using a combination of HbZ and plasma hepcidin with a better discriminatory ability for differentiating from WBC and CRP between children with KD and other FC. Using this predictor can assist front-line physicians to recognize and then provide early treatment for KD.

## 1. Introduction

Kawasaki disease (KD) is a febrile coronary vasculitis that primarily affects genetically susceptible younger children [1]. Even though the etiology of KD is not yet clearly known, this mysterious disease has been known as having factors of genetic susceptibility, infection, and immune overactivation [2,3,4,5]. Typical KD is identified by a prolonged fever for more than five days, with at least four of the following five presentations: diffuse superficial reddening of the lips and oral mucosa, nonexudative conjunctivitis, general polymorphous skin rashes, edema of the extremities or peeling of the fingertips, and cervical lymphadenopathy [2,4]. However, cases of incomplete KD, in which patients do not fulfill the KD definition, have been increasing [4]. Such cases have recently accounted for more than 20% of new-onset KD [4], especially in patients under six months of age [6]. 

In addition to the traditional diagnostic clinical criteria, KD patients may have a lots of nonspecific clinical presentations, like headaches; non-specific abdominal pain; hydrops of the gallbladder; erythematous rash at the bacillus Calmette–Guérin inoculation site; abnormal elevation of γ-glutamyl transferase, alanine aminotransferase, and aspartate aminotransferase; hypoalbuminemia, and decreased hemoglobin [7,8,9,10]. Notably, decreased hemoglobin in patients with KD is the most common character and is known as acute systemic inflammation or hemolysis [11,12,13]. Early and correct diagnosis of KD is necessary to assure promptly intravenous immunoglobulin (IVIG) treatment and prevent adverse coronary arterial aneurysms [14,15]. A previous dataset of 783 children (342 febrile children (FC) and 441 KD) showed that the hemoglobin level was one of the seven markers with the best diagnostic coefficients between the groups [16]. In a pediatric ICU, it was revealed that hemoglobin is a good predictor for distinguishing KD shock syndrome and toxic shock syndrome [17]. Liu et al. demonstrated that hemoglobin is also a predictor for distinguishing KD from sepsis in 227 KD and 193 sepsis [18]. Furthermore, we have demonstrated that hemoglobin levels are one of eight items of routine blood tests of a new score model for distinguishing KD from FC [19]. However, the hemoglobin cutoff levels in children vary by age [20]. As a result, a specific hemoglobin value cannot easily be clinically applied. In our previous study, we showed that IL-6 and plasma hepcidin levels were increased in KD patients compared to FC [21]. Kossiva et al. showed that plasma hepcidin and serum ferritin levels were significantly increased, whereas free iron was significantly more decreased in children with bacterial infection than with viral infection [22]. Hepcidin is the master in regulating iron metabolism and the key to the acute inflammation of anemia [23,24]. Of note, higher hepcidin in the KD before IVIG treatment is associated with resistance to IVIG [12] and significantly associated with the hospital stay of KD patients [25]. In the current study, we aim to develop a new model that combines the child hemoglobin-for-age z-score (HbZ) and plasma hepcidin without any clinical diagnostic criteria for differentiating between KD and FC. 

## 2. Materials and Methods

### 2.1. Study Population

This was a case-control study, in which we recruited 115 patients with KD and 104 with FC during 2012–2017. All patients with KD fulfilled the diagnostic criteria [26] and received IVIG treatment in the hospital. The details of typical and incomplete KD were explained in our previous paper [19]. All patients accepted at least one regimen of 2 g/kg IVIG under current practice guidelines [26]. We collected blood samples from patients with KD within 24 h before IVIG therapy. There were 54 airway, 11 gastrointestinal tract, 7 urinary tract, and 32 other infections patients in FC. Among them, there were 12 (11.5%) bacterial culture-proved cases. Informed consent was received from the parents or guardians of all patients. This study was authorized by our hospital’s Institutional Review Board (#201601023B0).

### 2.2. Quantification of Plasma Hepcidin by Enzyme-Linked Immunosorbent Assay

We gauged plasma hepcidin in 115 KD and 104 FC by commercial assays (# S-1337, Bachem Bioscience, St. Helens, UK). The full methodology was mentioned in previous studies [9,21,27]. 

### 2.3. Support Vector Machine 

A support vector machine (SVM) is an algorithm of machine learning that deals with binary classification problems and is often used in clinical biomedical studies [28,29,30]. In the current study, we used the lib-SVM module to gain a classification. To prevent selection bias, the person who performed the patients’ coding differed from that who analyzed the SVM. In the beginning, the training set (70%), which was composed of both positive and negative cases, was input into the SVM in a 5-fold cross-validation manner to obtain a model of classification. The optimal SVM parameter gamma and cost were coded as one and two, respectively. Next, we input the blind set (30%) into the SVM with the model of classification. Thereafter, the SVM reported whether this unknown case was positive or negative.

### 2.4. Statistical Analysis

Data in this study are presented as the mean ± standard error. For continuous variables, we adopted the independent *t*-test to compare the differences between KD and FC. Hemoglobin references vary among ages [31,32], and we used the reference for hemoglobin versus age [32]. The z-score of hemoglobin was corrected for age, and the standard deviation was included to execute the calculation [33]. The z-score is positive if the value is higher than the mean and negative if it is lower. We used the area under the receiver operating characteristic (auROC) curve to differentiate the parameters used to predict KD or FC. The best possibility of a cut-off value was declared using Youden’s Index. Meanwhile, we used the univariate or multivariate logistic regression model to identify significant independent markers for KD. All statistical analyses were conducted using SPSS version 22.0 for Windows XP (SPSS, Inc., Chicago, IL, USA). The two-sided *p*-value less than 0.05 was considered statistically significant in this study.

## 3. Results

### 3.1. Significantly Higher Plasma Hepcidin and Lower HbZ in KD Compared to FC

**Figure 1:**
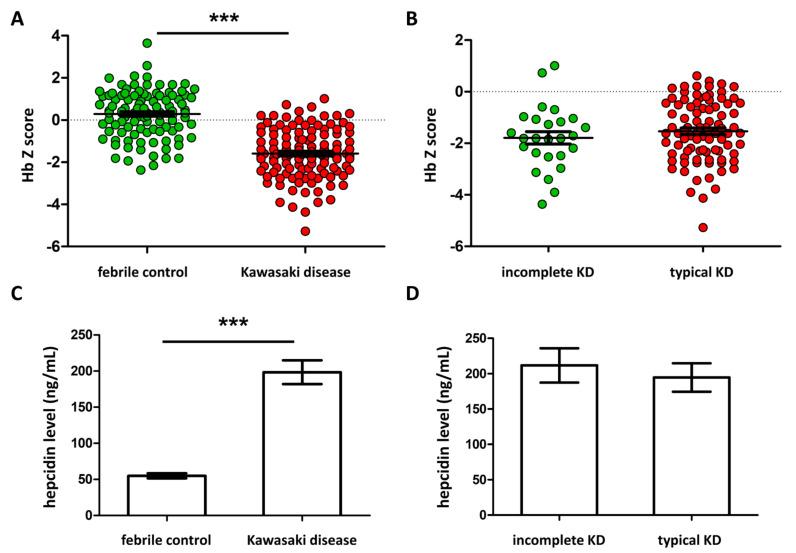
Comparison of the hemoglobin z-score and plasma hepcidin between patients with Kawasaki disease (KD) (N = 115) and febrile controls (FC) (N = 104). There are significantly lower HbZ (**A**) and higher plasma hepcidin (**C**) values in KD patients compared to FC. We did not find significant differences in either parameter between typical KD (N = 89) and incomplete KD (**B**,**D**). Asterisks denote significance (*** *p* < 0.001). Data are expressed as the mean ± standard error.

The clinical background of FC and KD patients are listed in Table 1. We found significant differences between KD and FC in gender, age, fever days, white blood count (WBC), C-reactive protein (CRP), and hemoglobin. Meanwhile, we did not find significant differences between typical and incomplete KD in these parameters, except fever duration before IVIG administration, which is consistent with the delayed diagnosis of incomplete KD. (Table 2) There were significantly higher plasma hepcidin and lower HbZ values in patients with KD compared to FC (all *p* < 0.001) (Figure 1A,C). We also did not observe any significant differences in either parameter between typical KD (N = 89) and incomplete KD (N = 26) (Figure 1B,D).

### 3.2. Significantly Better Preditor of the Combination of Plasma Hepcidin and HbZ in KD 

**Figure 2:**
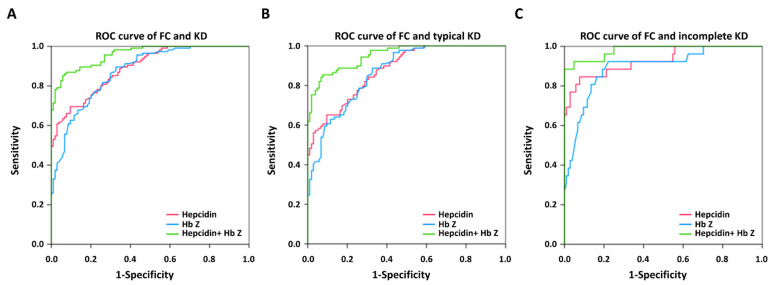
The area under the curve (AUC) of plasma hepcidin and hemoglobin z-score between (**A**) patients with Kawasaki disease (KD) (N = 115) and febrile controls (FC) (N = 104) was 0.959 (95% confidence interval = 0.926–0.978); (**B**) between typical KD patients (N = 89) and FC, it was 0.952 (95% confidence interval = 0.926–0.978); (**C**) between incomplete KD (N = 26) and FC, it was 0.981 (95% confidence interval = 0.956–1).

**Figure 3:**
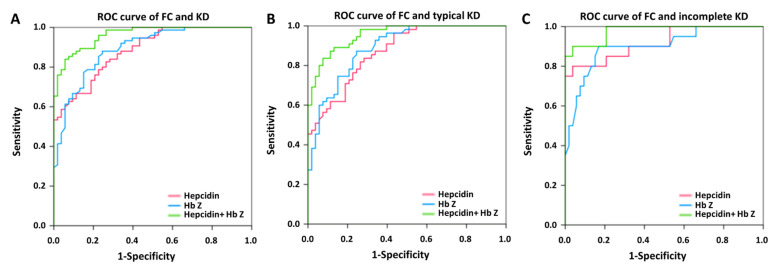
The area under the curve (AUC) of the plasma hepcidin and hemoglobin z-score between (**A**) boys with Kawasaki disease (KD) (N = 75) and febrile controls (FC) (N = 53) was 0.962 (95% confidence interval = 0.935–0.989); (**B**) between typical KD patients (N = 55) and FC, it was 0.955 (95% confidence interval = 0.922–0.988); (**C**) between incomplete KD (N = 20) and FC, it was 0.977 (95% confidence interval = 0.947–1).

**Figure 4:**
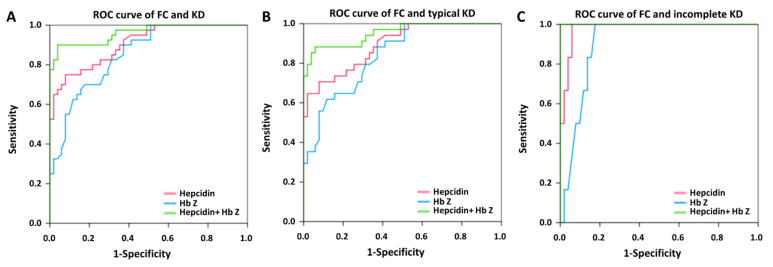
The area under the curve (AUC) of the plasma hepcidin and hemoglobin z-score between (**A**) girls with Kawasaki disease (KD) (N = 40) and febrile controls (FC) (N = 51) was 0.960 (95% confidence interval = 0.923–0.998); (**B**) between typical KD patients (N = 34) and FC, it was 0.952 (95% confidence interval = 0.908–0.996); (**C**) between incomplete KD (N = 6) and FC, it was 1 (95% confidence interval = 1–1).

We used multivariate logistic regression model analyses to identify predictors between KD and FC. Figure 2, Figure 3 and Figure 4 and Supplementary Figure S1 show the auROC curve plot of the selected candidate markers. Firstly, we analyzed the combination of WBC and CRP in the prediction of KD in FC. The auROC for KD and FC was 0.886, and the sensitivity and specificity were 80.5% and 81.4%, respectively (Supplementary Figure S1). Regarding typical and incomplete KD, the auROC for typical and incomplete KD versus FC was 0.894 and 0.860, respectively. Meanwhile, the sensitivity was 80.0% and 83.0%, as well as specificity being 81.4% and 76.5% between typical and incomplete KD versus FC, respectively. Then, we found that both HbZ and plasma hepcidin were independent predictors for distinguishing KD and FC with an auROC of 0.873 and 0.889, respectively (all *p* < 0.001) (Figure 2). In this analysis, the auROC for KD and FC was 0.959 (95% confidence interval = 0.937–0.981), and the sensitivity and specificity were 85.2% and 95.9%, respectively. Regarding typical and incomplete KD, the auROC for typical and incomplete KD versus FC was 0.952 (95% confidence interval = 0.926–0.978) and 0.981 (95% confidence interval = 0.956–1), respectively. Meanwhile, the sensitivity was 85.4% and 92.3%, as well as the specificity being 95.2, and 92.3% between typical and incomplete KD versus FC, respectively. In the light of KD being a male predominant disease. [1], we further divided KD patients into boys and girls. In consistency, we also gained good discrimination between boys or girls of KD and FC in auROC, sensitivity, and specificity (Figure 3 and Figure 4). From our results, the combination of HbZ and plasma hepcidin had a better discriminatory ability from WBC and CRP for KD, especially incomplete KD, and FC. 

### 3.3. SVM Classification 

We further used HbZ and plasma hepcidin as SVM vectors for each case. Among all the cases, 72 FC and 80 KD patients made up the training set (70% of total cases), while the other cohort of 32 FC and 35 KD patients formed the blind test set (70% of total cases). In the training set, the SVM classification model expressed 83.3% sensitivity and 88.0% specificity. Meanwhile, the blind cohort performed well, with 78.4% sensitivity and 100% specificity. Collectively, all data showed that the sensitivity and specificity were 81.7% and 91.3%, respectively. 

## 4. Discussion

In this study, our primary aim was to demonstrate that a novel predictor of a combination of HbZ and plasma hepcidin had a better discriminatory ability than WBC and CRP for distinguishing between patients with KD and FC without any of the clinical diagnostic criteria of KD. Overall, the sensitivity and specificity were 85.2% and 95.9% and 81.7% and 91.3% by multivariate logistic regression and SVM, respectively. Inconsistent with our previous study of KD incidence in Taiwan [1], we found males that predominated in KD in this study. Moreover, we also showed that both plasma hepcidin and HbZ could make a clear distinction between incomplete KD and FC. Indeed, the timing of the diagnosis of incomplete KD is the greatest challenge for front-line pediatricians [34,35]. In our previous report, we developed an SVM classification model of epigenetic hypomethylation of target CpG sites on the HAMP promoter and plasma hepcidin, which could accurately serve as KD biomarkers [27]. Nevertheless, the pyrosequencing assay for the HAMP promoter is currently too time-consuming and expensive for a front-line clinical application. Mounting evidence has revealed that anemia is a common characteristic found in KD patients [11,36,37,38]. In the past, we have demonstrated and reviewed that the common presentation of anemia is directly related to the significant upregulation of hepcidin expression in KD patients [9,21,27,39]. Hemoglobin levels were decreased and hepcidin levels were increased in KD patients before IVIG administration compared to in the febrile controls [9]. Within three days after completing IVIG therapy, both levels of plasma hepcidin and hemoglobin significantly decreased. The hemoglobin levels showed a gradual increase three weeks after the IVIG therapy. Therefore, we used HbZ and hepcidin instead of a pyrosequencing assay of HAMP and hepcidin for the HAMP promoter to achieve a simpler way to distinguish KD and FC. Furthermore, we also found that plasma hepcidin levels in KD patients correlated with the length of the hospital stay [40].

Clinical studies have revealed the relevance between sepsis and hepcidin, which has been helpfully used to evaluate the outcome of critical patients [41,42,43]. Septicemia can result in a decrease in serum iron and be related to high hepcidin levels [44]. We have consistently found that plasma hepcidin levels have a superior predictive value in distinguishing bacterial from viral infections, particularly in bacterial enteritis and urinary tract infections in FC, and are correlated to CRP levels [45]. Furthermore, Olinder et al. showed that patients with septic shock acquiring appropriate antibiotic treatment demonstrated a rapid and prompt decline of hepcidin within the first 24 h [46]. In addition, plasma hepcidin levels have a good predictive value for the 28-day ICU mortality of septic patients [47] and can reflect bacteremia in critical patients [48]. Recently, Peng et al. have found higher hepcidin and ferritin levels and lower iron levels in severe COVID-19 patients [49]. Meanwhile, the report by Delaye et al. shows that higher hepcidin levels are critical in patients with COVID-19 compared to in non-COVID-19 patients with similar CRP levels [50]. Growing evidence has revealed that multisystem inflammatory syndrome associated (MIS-C) with COVID-19 in children shares many clinical features [51] and inflammatory responses with KD [52]. Until now, there is no result of hepcidin in MIS-C, and it is worth further research.

Hemoglobin levels are decreased in FC [20], and anemia, mostly moderate, is a common presentation in children with bacterial meningitis, and it is also associated with poor outcomes [53]. Shi et al. found that children in ICU with lower hemoglobin levels have higher mortality in severe community-acquired pneumonia [54]. KD primarily affects children younger than five years old [55], and normal ranges for hemoglobin vary with an age under five-years-old [56]. Therefore, for front-line clinicians, an absolute value of hemoglobin is hard to apply to distinguish KD from FC. In current study, we also discovered significant differences in HbZ between KD and FC, which will be more straightforward for clinical application. Furthermore, the negative value of HbZ in KD (around −2) reflects a profound active inflammation in KD. 

Currently, KD is only diagnosed by clinical presentations. However, these clinical presentations are not often present at the same time or in the typical order of appearance [57]. Furthermore, only 70% to 80% of patients completely fulfill the KD criteria, and this percentage decreases in younger children [58]. Therefore, doctors who only suspect KD must perform repeat histories and careful examinations to timeously diagnose KD in FC. Incomplete KD poses an even bigger challenge for pediatricians [7]. As a result, numerous algorithms by routine laboratory parameters without clinical presentation for distinguishing KD and FC have been developed. In 2021, we demonstrated a novel score system with parameters of routine laboratory examination in pediatric emergency departments with good discriminatory ability for FC and KD patients; the overall sensitivity, specificity, and accuracy were 0.798, 0.847, and 0.844, respectively [19]. Other groups have also developed laboratory-only diagnostic models for KD, with a sensitivity and specificity of around 75–85% and 60–81%, respectively [16,59,60]. Our previous findings also showed that eosinophilia would be a vital predictive factor for patients with KD [33,61] and was associated with the failure of IVIG administration and coronary arterial formation [38,62]. Meanwhile, it was shown that higher procalcitonin levels occur in KD than in COVID-19 children [63] and septic children [18]. However, in a meta-analysis study, the overall diagnostic specificity and sensitivity of procalcitonin for KD were 0.78 and 0.73, respectively, and it concluded that procalcitonin is not a particularly useful diagnostic test for KD [64]. Prohormone brain natriuretic peptide also has a high diagnostic value for identifying KD in febrile children and the prediction of the IVIG response in KD [65,66]. Taken together, a combination of multiple significant biomarkers can improve the prediction of KD in FC.

This study has some limitations. Firstly, a selection bias may have been present because all data came from a single medical center and a limited number of cases. Although we obtained HbZ from the hemoglobin levels versus age from a reference [33], in the future, clinical samples of hemoglobin from a larger febrile population should be obtained to verify our findings. 

## 5. Conclusions 

Taken together, these findings highlight the significant change in HbZ levels in KD and FC. The combination of HbZ and plasma hepcidin had a good discriminatory ability and could serve as a predicting biomarker for KD, especially incomplete KD. 

## Figures and Tables

**Table 1:** Clinical characteristics between Kawasaki disease and febrile controls.

Characteristic	Febrile Controls (n = 104)	KD (n = 115)	*p*-Value
Male gender, n (%)	53 (51.0)	75 (65.2)	0.033
Age (year)	2.3 ± 0.2	1.8 ± 0.1	0.023
Fever duration (day)	4.5 ± 0.3	7.1 ± 0.2	<0.001
White blood cells (1000/uL)	9.8 ± 0.5	13.8 ± 0.5	<0.001
Hemoglobin (g/dL)	12.1 ± 0.1	11.1 ± 0.1	<0.001
C-reactive protein	20.0 ± 2.4	92.2 ± 6.6	<0.001

KD, Kawasaki disease. data expressed as mean ± standard error of mean.

**Table 2:** Clinical characteristics between patients with typical and incomplete Kawasaki disease.

Characteristic	Typical KD (n = 89)	Incomplete KD (n = 26)	*p*-Value
Male gender, n (%)	55 (61.8)	20 (76.9)	0.170
Age (year)	1.7 ± 0.1	1.8 ± 0.4	0.815
Fever duration (day)	6.9 ± 0.2	8.1 ± 0.7	0.031
White blood cell (1000/uL)	13.6 ± 0.5	14.5 ± 1.3	0.529
Hemoglobin (g/dL)	11.1 ± 0.1	10.8 ± 0.2	0.200
C-reactive protein (mg/L)	96.2 ± 7.9	78.2 ± 11.6	0.263

KD, Kawasaki disease. data expressed as mean ± standard error of mean.

## Data Availability

The datasets generated and analyzed during the current study are not publicly available due to strict ethical regulations of information privacy but are available from the corresponding author, Ying-Hsien, Huang upon reasonable request.

## Supplementary Materials

The following supporting information can be downloaded at: https://www.mdpi.com/article/10.3390/children9060913/s1, Figure S1: The area under the curve AUC of white blood cell and c reactive protein between (A) Kawasaki disease (KD) patients (N = 115) and febrile controls (FC) (N =104).
