# Supplementary material for: Combination of Hemoglobin-for-Age Z-Score and Plasma Hepcidin Identified as a Novel Predictor for Kawasaki Disease
Source: Children (Basel). 2022 Jun 18;9(6):913. doi: 10.3390/children9060913 (PMC9222120; doi:10.3390/children9060913)
Supplement: Supplementary file 1 [file children-09-00913-s001.zip › children-1743279-supplementary.pdf]

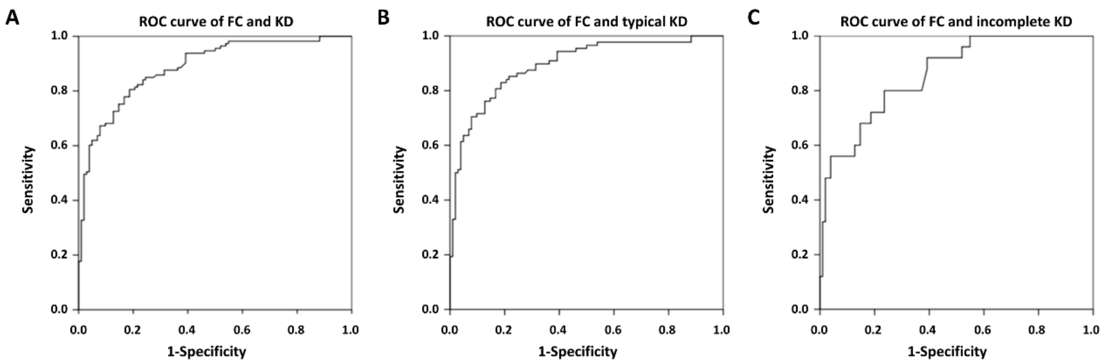

Supplementary Figure S1. The area under the curve AUC of white blood cell and c reactive protein between (**A**) Kawasaki disease (KD) patients (N = 115) and febrile controls (FC) (N =104) is 0.886 (95% confidence interval = 0.843-0.929); (**B**) typical KD patients (N = 89) and FC is 0.894 (95% confidence interval = 0.848-0.940); (**C**) incomplete KD (N = 26) is 0.860. (95% confidence interval = 0.783-0.936).
